# Whole-transcriptome analyses of sheep embryonic testicular cells infected with the bluetongue virus

**DOI:** 10.3389/fimmu.2022.1053059

**Published:** 2022-12-01

**Authors:** Danfeng Lu, Zhuoyue Li, Pei Zhu, Zhenxing Yang, Heng Yang, Zhanhong Li, Huachun Li, Zhuoran Li

**Affiliations:** ^1^ School of Medicine, Kunming University, Kunming, Yunnan, China; ^2^ School of Medicine and Pharmacy, Ocean University of China, Qingdao, Shandong, China; ^3^ Yunnan Tropical and Subtropical Animal Virus Diseases Laboratory, Yunnan Animal Science and Veterinary Institute, Kunming, Yunnan, China; ^4^ College of Agriculture and Life Sciences, Kunming University, Kunming, Yunnan, China

**Keywords:** Bluetongue virus, sheep embryonic testicular cells, RNA sequencing, ceRNA network, protein-protein interaction network

## Abstract

**Introduction:**

bluetongue virus (BTV) infection triggers dramatic and complex changes in the host's transcriptional profile to favor its own survival and reproduction. However, there is no whole-transcriptome study of susceptible animal cells with BTV infection, which impedes the in-depth and systematical understanding of the comprehensive characterization of BTV-host interactome, as well as BTV infection and pathogenic mechanisms.

**Methods:**

to systematically understand these changes, we performed whole-transcriptome sequencing in BTV serotype 1 (BTV-1)-infected and mock-infected sheep embryonic testicular cells, and subsequently conducted bioinformatics differential analyses.

**Results:**

there were 1504 differentially expressed mRNAs, 78 differentially expressed microRNAs, 872 differentially expressed long non-coding RNAs, and 59 differentially expressed circular RNAs identified in total. Annotation from the Gene Ontology, enrichment from the Kyoto Encyclopedia of Genes and Genomes, and construction of competing endogenous RNA networks revealed differentially expressed RNAs primarily related to virus-sensing and signaling transduction pathways, antiviral and immune responses, inflammation, and development and metabolism related pathways. Furthermore, a protein-protein interaction network analysis found that BTV may contribute to abnormal spermatogenesis by reducing steroid biosynthesis. Finally, real-time quantitative PCR and western blotting results showed that the expression trends of differentially expressed RNAs were consistent with the whole-transcriptome sequencing data.

**Discussion:**

this study provides more insights of comprehensive characterization of BTV-host interactome, and BTV infection and pathogenic mechanisms.

## Introduction

Bluetongue (BT) is one of the major arboviral diseases of ruminants, especially sheep, and manifests as various clinical symptoms in sick animals, ranging from subclinical symptoms to lethal hemorrhagic fever. It is estimated that BT is responsible for up to US$ 3 billion in annual economic losses in morbidity, mortality, production, reproduction, and animal-related trade restrictions (1, 2). Thus, the World Organization for Animal Health (OIE) listed BT as a notifiable disease. Bluetongue virus (BTV), the prototype virus of the *Orbivirus* genus within the *Reoviridae* family, is the pathogen of BT, which is mainly transmitted by *Culicoides* spp. midges. The genome of BTV is constituted by 10 segments of double-stranded RNAs (dsRNAs; Seg-1 to Seg-10) ranging in length from about 0.9 Kb to 4 Kb, encoding 7 structural proteins (VP1 to VP7) and 5 non-structural proteins (NS1 to NS4 and NS3A), in which VP2 and VP5 are the determinants of the viral serotypes (3). In total, 28 serotypes of BTV have been recognized globally, and additional novel serotypes have been identified continuously (4–6). There are 13 serotypes of BTV prevalent in China, including BTV-1, -2, -3, -4, -5, -7, -9, -12, -15, -16, -21, -24 and putative BTV-29 (5). The BTV-1 strain (Y863), in particular, caused the first recorded outbreak of BT in Shizong County, Yunnan <cp>Province</cp>, in 1979 (7). Furthermore, the Chinese national BTV surveillance program from 2013 to 2017 revealed that BTV was widely prevalent in Southern China, and Eastern-Western topotype reassorted strains were also derived through the genomic segments reassortment, potentially endangering animal husbandry safety production (5, 7). Therefore, an in-depth understanding of the pathogenesis of BTV and then exploring new strategies for BT prevention and control still rely on studying the interactions between BTV and the hosts.

Previous studies have pointed out that BTV replicates principally in endothelial cells and mononuclear phagocytic cells of sheep. However, it can also multiply efficiently in a variety of mammalian cell lines, such as primary sheep testicular (ST) cells, a cloned derivative baby hamster kidney (BSR) cells, and human cervical epithelial carcinoma (HeLa) cells, and subsequently release a large number of viral particles to trigger cytopathic effects (CPEs) in these infected cells, which is characterized by rounding, swelling, granular degeneration and detachment of cells (8–11). The infection and multiplication processes of viruses involve complex virus-host interactions. BTV’s success stems from its ability to actively manipulate antiviral defense, effectively subvert or take advantage of the host intracellular mechanisms, reform the environment of host cells by employing a set of virulence factors, and eventually produce virus-specific components. For example, BTV utilizes its nonstructural protein NS4 to antagonize the host interferon (IFN) response, downregulate the transcription levels of type I IFN (IFN-I) and IFN-stimulated genes (*ISGs*), and contribute to the virulence of BTV decisively (12). In addition, BTV regulated a series of host signaling pathways, such as mitogen-activated protein kinase (MAPK), phosphatidylinositide 3-kinase (PI3K)-serine/threonine kinase (Akt), and nuclear factor-kappa B (NF-κB) signaling pathways, by altering the expression profile of host microRNAs (miRNAs) (10).

Recently, lots of studies have reported that long noncoding RNAs (lncRNAs) and circular RNAs (circRNAs) function in the infection and replication processes of viruses through modifying the transcriptomic responses of hosts and participating in various cellular processes, including but not limited to antiviral immunity, metabolic pathways, and cellular apoptosis (13, 14). For instance, circRNAs serves as sponges of miRNAs and constitute an interactive competing endogenous RNA (ceRNA) network with miRNAs and mRNAs to modulate RNA transcription and protein production. Viruses destroy or utilize the networks, which can significantly influence the viral life cycle and pathogenicity (14). However, studies on the transcriptional profiles of BTV-infected cells have either been conducted in human cell lines or have only sequenced the miRNAs/mRNAs sector (10, 12, 15). There is no whole-transcriptome study of susceptible animal cells with BTV infection, which impedes the in-depth and systematical understanding of the comprehensive characterization of BTV-host interactome, as well as BTV infection and pathogenic mechanisms.

In this study, we performed whole-transcriptome sequencing in BTV-1-infected sheep embryonic testicular cells, conducted a series of bioinformatics analyses, clearly portrayed the RNAs expression profile underlying responses to BTV infection at a critical time point, and finally gained some insights into the basic molecular mechanisms of host-virus interactions.

## Materials and methods

### Cell culture and infection

Sheep embryonic testicular (OA3.Ts) cells were purchased from the Kunming Branch of the National Experimental Cell Resource Sharing Platform, Chinese Academy of Science. The cells were cultured in F12: DMEM =1: 1 medium supplemented with 10% FBS at 37 °C/5% CO_2_. OA3.Ts cells were seeded in 10 cm dishes (NEST, China), grown to approximately 80%~90% confluency, and inoculated with the BTV-1 (Y863 strain) with a multiplicity of infection (MOI) of 1. The supernatants were discarded after 1 hour of incubation. OA3.Ts cells were then maintained with F12: DMEM =1: 1 medium supplemented with 1% FBS. The uninfected OA3.Ts cells served as the mock-infected control. The CPEs were observed under a light microscope (Olympus, Japan) at 0, 24 and 48 h post-infection (hpi).

### RNA extraction and qualification

Total RNA from each sample was extracted with TRIzol reagent (Invitrogen, USA) according to the manufacturer’s instructions. The RNA amount and purity of each sample were quantified using NanoDrop ND-100 (NanoDrop, USA). The RNA integrity was assessed by Bioanalyzer 2100 and RNA 6000 Nano LabChip Kit (Agilent, USA) with RIN number >7.0 and confirmed by electrophoresis with denaturing agarose gel. The extracted RNA was stored at -80 °C until use.

### Library construction and sequencing

Poly(A) RNA was purified for two rounds from 2 μg total RNA using Dynabeads Oligo(dT)25-61005 (Thermo Fisher, USA) to construct mRNA library. Approximately 2 μg of total RNA was used to remove ribosomal RNA according to the manuscript of the Ribo-Zero Gold Kit (Epicentre Biotechnologies, USA) to construct lncRNA or circRNA library. The poly(A) RNA and the remaining RNA after ribosomal RNA removal were fragmented into small pieces using Magnesium RNA Fragmentation Module (NEB, USA). The cleaved RNA fragments were reverse-transcribed into cDNA by SuperScript II Reverse Transcriptase (Invitrogen), which was subsequently used to synthesize U-labeled second-stranded DNAs with *E. coli* DNA polymerase I (NEB), RNase H (NEB) and dUTP Solution (Thermo Fisher). An A-base was then added to the blunt ends of each strand, followed by the ligation of indexed adapters. Each adapter contained a T-base overhang for ligating the adapter to the A-tailed fragmented DNA. Next, single- or dual-index adapters were ligated to the fragments, and size selection was performed with AMPure XP beads (Beckman Coulter, USA). After the heat-labile UDG enzyme (NEB) treatment of the U-labeled second-stranded DNAs, the ligated products were amplified with PCR. The average insert size for the final cDNA library was 300 ± 50 bp.

Approximately 1 μg of total RNA was used to prepare the miRNA library following the protocol of TruSeq small RNA Sample Prep Kits (Illumina, USA). Briefly, the miRNA molecules were ligated to a 5’ adaptor and a 3’ adaptor by T4 RNA ligase (Promega, USA). Subsequently, the adaptor-ligated miRNAs were reverse transcribed into cDNA and PCR amplified. Finally, PCR products were purified and DNA fragments of 150 ± 10 bp (the length of small RNA inserts plus the 5’ and 3’ adaptors) were quantified following the Solexa sequencing protocol (Illumina). At last, the 2×150 bp paired-end sequencing for mRNA, lncRNA, and circRNA, and the 1×50 bp single-end sequencing for miRNA were performed on Illumina Novaseq 6000 and Illumina Hiseq2500 (LC-Bio Technology Co. Ltd., Hangzhou, China) following the vendor’s recommended protocol, respectively. The obtained sequence reads were deposited into the NCBI Gene Expression Omnibus (GEO) database and can be accessed *via* accession numbers GSE213637 and GSE213638.

### Bioinformatics analyses

Fastp (v0.14.1) was used to remove the mRNA, lncRNA and circRNA reads that contained adaptor contamination, low-quality bases and undetermined bases and verify the sequence quality (16). Hisat2 (v2.0.4) for mRNA, as well as Bowtie2 (v2.2.5) and Tophat2 (v2.0.13) for lncRNA were used to map reads to the genome of *Ovis aries* (GCA_000298735.1) (17–19). StringTie (v1.3.0) was used to assemble the mapped mRNA and lncRNA reads from each sample, and then the mRNA or lncRNA transcripts from all samples were merged to reconstruct a comprehensive transcript profile using GffCompare (20, 21). Subsequently, transcripts annotated as known mRNAs, known lncRNAs, and less than 200 nt in length were discarded. Coding Potential Calculator (CPC) software (v0.9-r2) and Coding-Non-Coding Index (CNCI) software (v2.0) were unutilized to predict transcripts with coding potential. All transcripts with CPC score <-1 and CNCI score <0 was removed. The remaining transcripts with class code (l, j, o, u, and x) were considered novel lncRNAs (22, 23). The expression levels of mRNAs and lncRNAs were calculated using fragments per kb per million reads (FPKM = [total exon fragments/mapped reads (millions) × exon length (Kb)]) (20).

Bowtie2 (v2.2.5) and Tophat2 (v2.0.13) for circRNA were used to map reads to the *Ovis aries* genome (GCA_000298735.1) (18, 19). The remaining unmapped circRNA reads were further mapped to the genome using Tophat-Fusion (v2.0.12) (24). As for mapped circRNA reads, they firstly were *de novo* assembled to circular using CIRCexploer (v1.1.10) and CircRNA Identifier (CIRI) (v2) (25–27). Then, back-splicing reads were identified in unmapped reads by Tophat-Fusion (v2.0.12) and CIRCexploer (v1.1.10) (24–26). All samples generated unique circular RNAs. Spliced reads per billion mapping (SRPBM) calculated circRNAs expression levels (SRPBM = number of back-spliced junction reads/number of mapped reads × 1,000,000,000).

The miRNA raw data were processed through an in-house program, ACGTA101-miR (v4.2) (LC Science, USA) to remove adapter dimers, junk, low complexities, common RNA families (rRNA, tRNA, snRNA, and snoRNA), repeats, and sequences <18 nt or >26 nt in length were filtered out using Rfam (v13.0) and Repbase Update (28, 29). The 18~26 nt unique reads were mapped to miRNA sequences in miRBase (v22.0) (30). Mapping was also performed on pre-miRNA against *Ovis aries* genomic data (GCA_000298735.1). The unique sequences that aligned to the known miRNA sequences in miRBase (v22.0) were identified as known miRNAs (30). The secondary structure of pre-miRNAs was presented as a hairpin, including 5p- and 3p- derived miRNA. The unique sequences mapping to the other arm of the pre-miRNA sequences that were not annotated in the miRBase (v22.0) were considered to be 5p- or 3p-derived miRNA candidates (30). In order to find candidate novel miRNAs, the remaining unmapped sequences were compared to the *Ovis aries* genomic sequences (GCA_000298735.1). In order to identify the results of putative miRNAs of *Ovis aries*, all the obtained miRNAs were used to predict the secondary structures using RNAfold software (31).

R package edgeR (v3.14.0) was used to analyze the differentially expressed RNAs (dif-RNAs) (32). The thresholds of |log_2_ fold change (FC)| ≥1.0 and *p*-value <0.05 were used to filter out differentially expressed mRNAs (dif-mRNAs), differentially expressed lncRNAs (dif-lncRNAs), and differentially expressed circRNAs (dif-circRNAs), while only the comparisons with *p*-value <0.05 were considered differentially expressed miRNAs (dif-miRNAs) based on normalized deep-sequencing counts. Hierarchical clustering and volcano plot were drawn on the dif-RNAs using the pheatmap R package (v1.0.12) and ggplot2 R package (v3.3.6), respectively (33, 34).

### Target gene prediction and functional analyses

To further explore the function of dif-RNAs, potential *cis*-target mRNAs of dif-lncRNAs were predicted (35). The *cis*-targets of dif-lncRNAs were predicted using Blast2GO (v4.0.7) (36). The mRNAs in the dif-lncRNAs’ upstream or downstream 100 Kb regions were considered potential *cis-*targets.

The target mRNAs for each dif-miRNAs were predicted using the TargetScan (v5.0) and miRanda (v3.3a) algorithms (37). TargetScan score ≥50 and miRanda energy <-10 were set as thresholds for screening. Finally, the data predicted by both algorithms were combined and the overlaps were analyzed.

Gene Ontology (GO) annotations were performed on dif-mRNAs, and target or host mRNAs of dif-lncRNAs, dif-miRNAs, and dif-circRNAs using gene ontology resource (38). In addition, the Kyoto Encyclopedia of Genes and Genomes (KEGG) enrichment analyses were conducted to understand high-level functions and utilities of biological systems (39). GO bubble diagrams and KEGG scatter diagrams were visualized using the GOplot R package (v1.0.2) and ggplot2 R package (v3.3.6), and all terms with *p*-value <0.05 were considered significantly (34, 40).

### ceRNA networks construction

In constructing the lncRNA- and circRNA-miRNA-mRNA axes, the most meaningfully interacting pairs were selected and presented according to the following criteria: 1) Among miRNA-mRNA interaction pairs, dif-miRNAs and dif-mRNAs were selected when their |log_2_ FC| values were ≥1.5 and ≥2.0, respectively. At the same time, the pairs should comply with a standard of TargeScan score ≥50 and miRanda energy <-10; 2) For miRNA-lncRNA interaction pairs, the values of |log_2_ FC| were the same as those of miRNA-mRNA pairs; while the TargeScan score ≥80 and miRanda energy <-20 were set as a further filter criterion; 3) As for miRNA-circRNA interaction pairs, the thresholds of |log_2_ FC|, TargetScan score, and miRanda energy were consistent with those of miRNA-mRNA pairs. Subsequently, the lncRNA- and circRNA-miRNA-mRNA axes were constructed with miRNAs as the central nodes, and ceRNA networks were built and visually displayed using the ggalluvial R package (v0.12.3) based on these axes (41). The differential transcripts of mRNAs involved in the ceRNA networks were performed GO and KEGG analyses. These results were graphically displayed with the GOplot R package (v1.0.2) and ggplot2 R package (v3.3.6), and all terms with *p*-value <0.05 were considered significantly (34, 40).

### Protein-protein interaction (PPI) network and module analysis of dif-mRNAs

The interactions between dif-mRNAs encoded proteins were analyzed using the Search Tool for the Retrieval of Interacting Genes/Proteins (STRING) online database (v11.5) (42). The species was set as *Ovis aries* with the interaction score >0.4 selected as the cut-off value. Subsequently, the PPI network was visualized utilizing Cytoscape software (v3.8.2) (43). The Cytoscape’s Molecular Complex Detection (MCODE) plugin was applied to extract densely connected modules from the PPI network, with degree cut-off = 2, node score cut-off = 0.2, K-score = 2, and max depth = 100. The downregulated and upregulated mRNAs involved in the PPI network were performed GO and KEGG enrichment and visualized using the ggplot2 R package (v3.3.6), respectively (34). All terms with *p*-value <0.05 were considered significantly.

### Real-time quantitative PCR (qRT-PCR)

Real-time qRT-PCR was conducted on an ABI 7500 fast system (Applied Biosystems, USA) using One Step PrimeScript Real Time RT-PCR Kit (Takara Biomedical Technology, China) with extracted total RNA from OA3.Ts cells served as templates to verify the successful infection of BTV-1 (Y863 strain) (44).

In order to validate the expression profile of dif-RNAs, real-time qRT-PCR was performed using PrimeScript RT reagent Kit with gDNA Eraser (Takara Biomedical Technology) and TB Green Premix Ex Taq II (Takara Biomedical Technology) for dif-mRNAs, dif-lncRNAs, and dif-circRNAs, as well as Mir-X miRNA First-Stranded Synthesis Kit (Takara Biomedical Technology) and Mir-X miRNA qRT-PCR TB Green Kit (Takara Biomedical Technology) for dif-miRNAs on an ABI 7500 fast system (Applied Biosystems) according to the manufacturer’s instructions. The specific primers were designed using Oligo 7 software or referred to as the reported primers. The primer sequences are shown in **Supplementary Table 1**. Transcriptional levels of the gene for *β-actin* and *U6* were determined to normalize total RNA input. Relative dif-RNAs expression was evaluated using the 2^-ΔΔCt^ method.

### Western blotting (WB)

The infected cells were washed with cold phosphate-buffered saline (PBS) and lysed for 30 min at 4°C in lysis buffer (Beyotime Biotechnology, China) containing protease inhibitor cocktails (BioTool, China). Total protein quantities of the sample supernatants were determined using TaKaRa BCA Protein Assay Kit (Takara Biomedical Technology). Equal amounts (20 μg) of each quantified cell proteins were separated on 10% SDS-PAGE and then transferred onto the PVDF membrane (Millipore, USA). After blocking with 5% bovine serum albumin (BSA) (Sangon Biotech, China) in PBS containing 0.1% Tween (Sangon Biotech), the blots were incubated with Rig-I (D33H10) Rabbit mAb (CST, USA), IκBα (L35A5) Mouse mAb (CST), IKKγ antibody (CST), Caspase-8 (D35G2) Rabbit mAb (CST), Anti-CTGF antibody (Abcam, UK), PIK3IP1 Ab (Affinity Biosciences, China), NS3 (33H7) mouse monoclonal antibody (Ingenasa, Spain), anti-β-tubulin mouse monoclonal antibody (Transgen Biotech, China), and Anti-beta Actin antibody (Abcam) at 4°C overnight, respectively. This was followed by incubation with horseradish peroxidase (HRP)-conjugated goat anti-mouse IgG antibody (Beyotime Biotechnology) or HRP-conjugated goat anti-rabbit IgG antibody (Beyotime Biotechnology). In addition, the immunoreactivity of blots with antibodies was visualized under ChemiScope 3300mini (Clinx, China) using the BeyoECL Plus Detection Kit (Beyotime Biotechnology). The relative optical densities of bands were assessed using ImageJ software (Bethesda, USA) and normalized according to optical densities of corresponding β-tubulin bands.

### Statistical analysis

Data were presented as mean ± SD with an error bar representing at least three independent experiments. In addition, the student’s t test was performed for statistical analysis with GraphPad Prism 7.0 software (GraphPad Software, USA). Symbols * and ** indicate that the difference between the indicated groups was significant (*p*-value <0.05) or very significant (*p*-value <0.01), respectively.

## Results

### Cells infection and harvest

**Figure 1:**
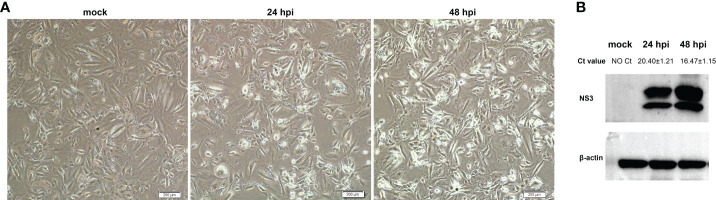
Validation of BTV-1 infection in OA3.Ts cells at 0, 24 and 48 dpi. **(A)** CPEs were observed in OA3.Ts cells infected with BTV-1 (Y863 strain) under microscope at 0, 24 and 48 hpi (×4 magnification). Scale bar, 200 μM. **(B)** qRT-PCR and WB were also used to verify the successful infection of BTV-1 (Y863 strain) in OA3.Ts cells. The infection status of BTV-1-infected- and mock-infected groups at different time points was represented by mean Ct values ± SD from three independent experiments. NS3 was detected as control for successful infection, whereas β-actin served as an internal control.

OA3.Ts cells were infected with BTV-1 at a MOI of 1, and the infection was confirmed by observing CPEs and monitoring virus replication by qRT-PCR and WB at 0, 24 and 48 hpi. As it showed in **Figure 1A**, no CPE was observed in mock-infected OA3.Ts cells, while the pathological cellular state in the BTV-1-infected group, with cells shrinkage and rounding, could begin to be recognized at 24 hpi, and the CPEs were more obvious at 48 hpi. The results of virus replication were displayed in the form of cycle threshold (Ct) values and viral NS3 protein bands of WB (**Figure 1B**). The gradual increase in viral replication from 24 hpi to 48 hpi was reflected in decreasing Ct values and increased NS3 protein bands, indicating the development of persistent infection. The OA3.Ts cells at 24 hpi were harvested for whole-transcriptome sequencing.

### Differential expression profiles analyses

**Figure 2:**
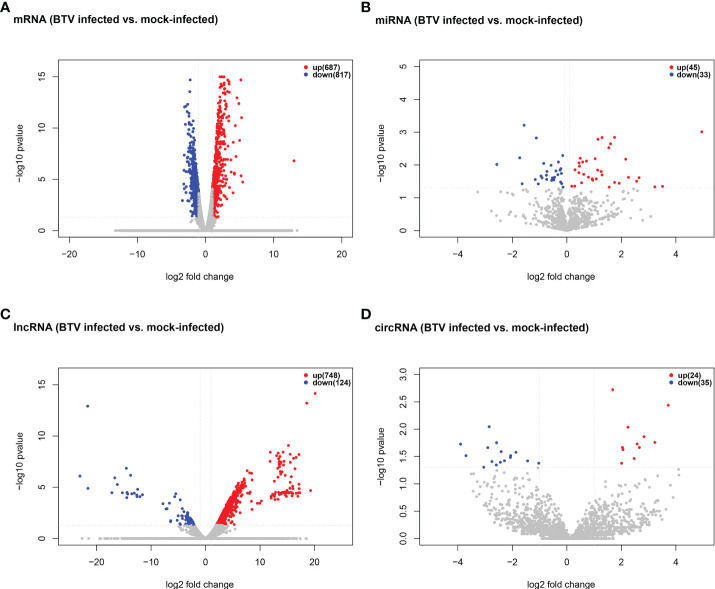
Volcano plots of **(A)** dif-mRNAs, **(B)** dif-miRNAs, **(C)** dif-lncRNAs, and **(D)** dif-circRNAs between BTV-1-infected and mock-infected OA3.Ts cells. Red and blue dots represented upregulated and downregulated dif-RNA, respectively. The total numbers of upregulated and downregulated dif-RNAs were marked in the upper right corner of each figure.

**Figure 3:**
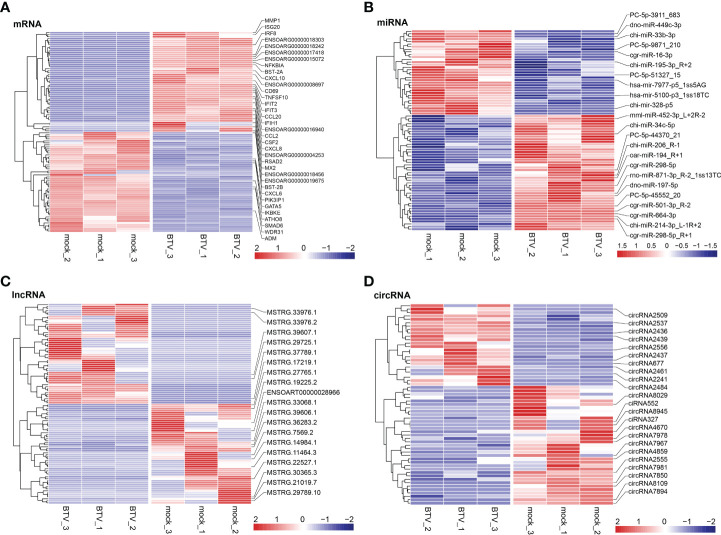
Hierarchical cluster analyses of **(A)** dif-mRNAs, **(B)** dif-miRNAs, **(C)** dif-lncRNAs, and **(D)** dif-circRNAs between BTV-1-infected and mock-infected OA3.Ts cells. The top 50 upregulated and downregulated dif-mRNAs and dif-lncRNAs, as well as all dif-miRNAs and dif-circRNAs were selected and displayed in the heatmaps. The color of the color blocks from blue to red represented the dif-RNAs expression levels from downregulated to upregulated. Several important dif-RNAs were labeled to the right of the corresponding heatmaps.

Whole-transcriptome sequencing was applied to profile the expression of mRNAs, miRNAs, lncRNAs, and circRNAs in BTV-1-infected OA3.Ts cells. Overall, 1504 dif-mRNAs, containing 687 upregulated and 817 downregulated mRNAs, were identified in the infected group compared with the mock-infected group (**Figure 2A** and **Supplementary Table 2**). According to the screening criteria, 78 dif-miRNAs were determined, of which 45 were transcriptionally increased and 33 were decreased (**Figure 2B** and **Supplementary Table 3**). A total of 872 dif-lncRNAs, consisting of 863 novel lncRNAs and 9 known lncRNAs, were obtained, including 748 upregulated and 124 downregulated lncRNAs (**Figure 2C** and **Supplementary Table 4**). In addition, the expression levels of 24 circRNAs were increased, and 35 circRNAs were decreased among 59 dif-circRNAs, (**Figure 2D** and **Supplementary Table 5**). Hierarchical clustering was performed to generate heatmaps of dif-RNAs, from which it was found that the infected samples could be significantly separated from the mock-infected samples (**Figures 3A–D**). Moreover, consistent with the previous study conducted in BTV-16-infected peripheral blood mononuclear cells (PBMCs), our study similarly obtained more than 1000 dif-mRNAs; some of the 1504 dif-mRNAs involved in antiviral activities and immune responses also presented high expression levels, including *ISG20*, IFN-induced protein with tetratricopeptide repeats-1 (*IFIT1*) and *IFIT2* (**Figure 3A** and **Supplementary Table 2**) (15). Although the number of identified dif-miRNAs was limited, the expression patterns of certain dif-miRNAs were similar to the results of microRNA sequencing in BTV-1 (GS/11 strain)-infected ST cells, such as chi-miR-33b-3p, chi-miR-34c-5p and oar-miR-194_R+1 (**Figure 3B** and **Supplementary Table 3**) (10). All these data indicated that the results of the differential expression analyses were reliable.

### Functional enrichment analyses of dif-RNAs

To better understand the potential roles of host factors in BTV infection, all dif-RNAs were subjected to GO term classification statistics from the biological processes (BP), cellular components (CC), and molecular function (MF) ontologies.

**Figure 4:**
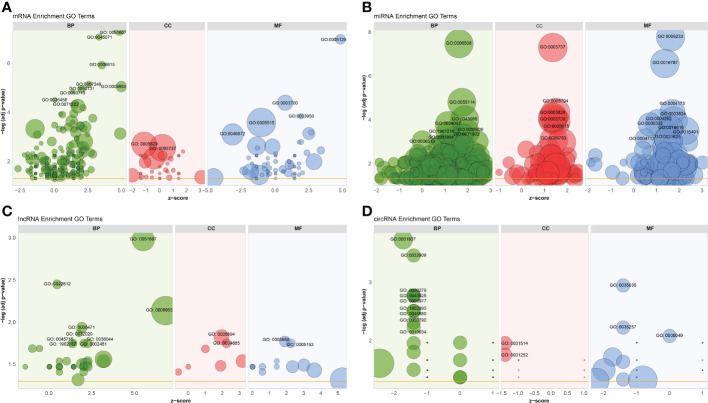
GO annotations of **(A)** dif-mRNAs, target mRNAs of **(B)** dif-miRNAs, **(C)** dif-lncRNAs, and **(D)** host mRNAs of dif-circRNAs under BP, CC, and MF ontologies, respectively. Z-score was equal to the total number of transcriptional increased dif-mRNAs minus the total number of decreased dif-mRNAs and then divided by the square root of the total number of dif-mRNAs in a certain GO term and was used to measure whether the term was upregulated or downregulated overall. The size of the solid circulars represented the number of enriched dif-mRNAs under specific GO terms. GO: 0051607: defense response to virus, GO: 0045071: negative regulation of viral genome replication, GO: 0009615: response to virus, GO: 0007249: I-kappaB kinase/NF-kappaB signaling, GO: 0006955: immune response, GO: 0050731: positive regulation of peptidyl-tyrosine phosphorylation; GO: 0050713: negative regulation of interleukin-1 beta secretion, GO: 0035458: cellular response to interferon-beta, GO: 0071222: cellular response to lipopolysaccharide, GO: 0005829: cytosol, GO: 0005737: cytoplasm, GO: 0005125: cytokine activity, GO: 0003700: DNA-binding transcription factor activity, GO: 0003950: NAD+ADP-ribosyltransferase activity, GO: 0005515: protein binding, GO: 0046872: metal ion binding; GO: 0006508: proteolysis, GO: 0055114: oxidation-reduction process, GO: 0043066: negative regulation of apoptotic process, GO: 0034097: response to cytokine, GO: 0006606: protein import into nucleus, GO:1901216: positive regulation of neuron death, GO: 0071902: positive regulation of protein serine/threonine kinase activity, GO: 0001934: positive regulation of protein phosphorylation, GO: 0006513: protein monoubiquitination, GO: 0005794: Golgi apparatus, GO: 0005739: mitochondrion, GO: 0005615: extracellular space, GO: 0005783: endoplasmic reticulum, GO: 0008233: peptidase activity, GO: 0016787: hydrolase activity, GO: 0004175: endopeptidase activity, GO: 0003824: catalytic activity, GO: 0004252: serine-type endopeptidase activity, GO: 0030332: cyclin binding, GO: 0016616: oxidoreductase activity, acting on the CH-OH group of donors, NAD or NADP as acceptor, GO: 0016491: oxidoreductase activity, GO: 0004712: protein serine/threonine/tyrosine kinase activity, GO: 0031625: ubiquitin protein ligase binding; GO: 0022612: gland morphogenesis, GO: 0006471: protein ADP-ribosylation, GO: 0032020: ISG15-protein conjugation, GO: 0038044: transforming growth factor-beta secretion, GO: 0002481: antigen processing and presentation of exogenous protein antigen *via* MHC class Ib, TAP-dependent, GO:1902037: negative regulation of hematopoietic stem cell differentiation, GO: 0045715: negative regulation of low-density lipoprotein particle receptor biosynthetic process, GO: 0005694: chromosome, GO: 0034685: integrin alphav-beta6 complex, GO: 0005153: interleukin-8 receptor binding; GO: 0001837: epithelial to mesenchymal transition, GO: 0032909: regulation of transforming growth factor beta2 production, GO: 0030279: negative regulation of ossification, GO: 0043525: positive regulation of neuron apoptotic process, GO: 0005977: glycogen metabolic process, GO:1902895: positive regulation of pri-miRNA transcription by RNA polymerase II, GO: 0045880: positive regulation of smoothened signaling pathway, GO: 0050790: regulation of catalytic activity, GO: 0010634: positive regulation of epithelial cell migration, GO: 0031514: motile cilium, GO: 0031252: cell leading edge, GO: 0035035: histone acetyltransferase binding, GO: 0035257: nuclear hormone receptor binding, GO: 0000049: tRNA binding.

Similar to the research performed in BTV-16-infected sheep PBMCs, BTV-1 infection induced significant enrichment of defense response to virus (GO: 0051607) and immune response (GO: 0006955) under BP ontology, and protein binding (GO: 0005515) and metal ion binding (GO: 0046872) under MF ontology in OA3.Ts cells (**Figure 4A** and **Supplementary Table 2**) (15). Compared with the mock-infected group, in addition to well-known antiviral genes, such as *IFITs* and radical S-adenosyl methionine domain containing 2 (*RSAD2*), several antiluteolysin genes belonging to the IFN-I family and relevant to preventing pregnant ewes from luteolytic, for example *ENSOARG00000008675*, *ENSOARG00000008697*, and *ENSOARG00000008791*, also transcriptionally increased under cytokine activity (GO: 0005125) and defense response to virus (GO: 0051607) terms, indicating that these antiluteolysin genes potentially contribute to host antiviral responses (**Figure 3A** and **Supplementary Table 2**) (15, 45). The other dif-mRNAs in comparison with mock-infected and infected samples were mostly associated with negative regulation of viral genome replication (GO: 0045071), response to virus (GO: 0009615), I kappa B kinase (IKK)/nuclear factor-kappa B (NF-κB) signaling (GO: 0007249), cytokine activity (GO: 0005125) and DNA-binding transcription factor activity (GO: 0003700) under BP and MF ontologies, respectively (**Figure 4A** and **Supplementary Table 2**). Interestingly, the key activators of the NF-κB signaling pathway, which had been reported to be activated by BTV infection, were downregulated, for instance, an inhibitor of NF-κB kinase subunit ϵ (*IKBKE*) and *IKBKG* (also known as NF-κB essential modulator, *NEMO*). While inhibitors of NF-κB signaling pathway, such as NF-κB inhibitor α (*NFKBIA* encoding IκBα) and *NFKBIB*, were upregulated significantly. These transcriptional alternations were accompanied by increases in the expression of tumor necrosis factor (TNF) receptor-associated factor family member associated NF-κB activator (*TANK*), NF-κB functional subunit *REL*, NF-κB p105 subunit (*NFKB1*), and *RELB* (**Figure 3A** and **Supplementary Table 2**) (11, 46–48). A similar phenomenon was also found in the BTV-16-infected sheep PBMCs, for example, a significant decrease in *IKBKE* transcription (15). These results indicated that canonical and non-canonical NF-κB pathways might play a more complicated and changeable role in BTV infection and pathogenic processes.

MiRNAs regulate gene expression at the post-transcriptional level through interactions between their seed sequences and the target mRNAs (49). The dif-miRNAs target genes in this study were primarily enriched in the BP ontology of proteolysis (GO: 0006508) and oxidation-reduction process (GO: 0055114), the CC ontology of cytoplasm (GO: 0005737), and the MF ontology of peptidase activity (GO: 0008233), hydrolase activity (GO: 0016787) and endopeptidase activity (GO: 0004175) (**Figure 4B** and **Supplementary Table 6**). By analyzing the GO classification of dif-miRNA target genes in BTV-1-infected ST cells, it was found that under BP and MF ontologies, the enriched GO terms involved in both our current and previous studies are related to metabolic process and catalytic activity, respectively. In contrast, under CC ontology, our study is mainly associated with cytoplasm rather than cell and cell part (10). According to the definition of lncRNA function mode and the prediction of target genes, 622 dif-lncRNAs might influence the expression or chromatin state of 1077 of their nearby genes in *cis* (**Supplementary Table 7**) (35). The target genes mainly involved in the BP ontology of defense response to virus (GO: 0051607), gland morphogenesis (GO: 0022612), immune response (GO: 0006955), protein adenosine-diphosphate (ADP)-ribosylation (GO: 0006471) and IFN-stimulated gene 15 (ISG15)-protein conjugation (GO: 0032020), and the CC ontology of chromosome (GO: 0005694) (**Figure 4C** and **Supplementary Table 7**). For instance, two significantly elevated dif-lncRNAs, MSTRG.33976.1 and MSTRG.33976.2, were probably responsible for the several folds upregulation of their target gene C-X-C motif chemokine 8 (*CXCL8*, also known as *IL-8*) in immune response (GO: 0006955) term (**Figure 3A, C**; **Figure 4C** and **Supplementary Table 7**). Among 59 dif-circRNAs, 38, 3, and 18 were back-spliced from exons, introns, and intergenic RNAs, respectively (**Supplementary Table 5**). The linear transcripts of the 41 dif-circRNAs were identified. Following that, the corresponding transcripts were subjected to GO enrichment analysis. These linear cognates were significantly assigned to BP and MF ontologies, including epithelial to mesenchymal transition (GO: 0001837), regulation of transforming growth factor β (TGF-β)-2 production (GO: 0032909), negative regulation of ossification (GO: 0030279), positive regulation of neuron apoptotic process (GO: 0043525), glycogen metabolic process (GO: 0005977), and histone acetyltransferase binding (GO: 0035035) (**Figure 4D** and **Supplementary Table 8**). These results suggested that dif-RNAs induced by BTV-1 infection are not only related to antiviral and immune responses of the host, but also participate in the development and metabolism processes.

### Enrichment analyses of dif-RNAs

It is well known that signaling pathway analyses conduce to better understanding of the biological functions of genes. KEGG pathway enrichment analyses of dif-RNAs can be used to determine the biochemical metabolic and signal transduction pathways, further facilitating the exploration of host-virus interactions.

**Figure 5:**
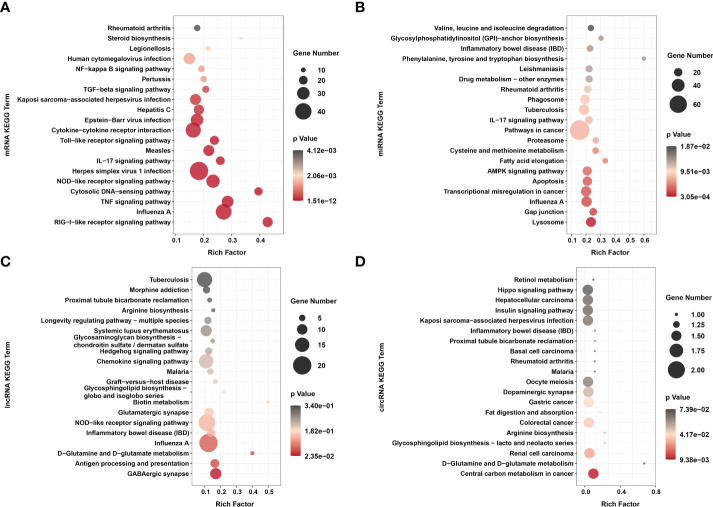
KEGG enrichments of **(A)** dif-mRNAs, target mRNAs of **(B)** dif-miRNAs, **(C)** dif-lncRNAs, and **(D)** host mRNAs of dif-circRNAs. Top 20 KEGG pathways were shown. Rich factor indicated the ratio of dif-mRNAs enriched to the specific pathway. The size and the color of the solid circulars represented the number of enriched dif-mRNAs and significance of the enrichment, respectively.

KEGG analysis showed that unlike the results of previous research, dif-mRNAs lacked primary enrichment in Ras, MAPK, Janus tyrosine kinase (JAK)-signal transducer and activator of transcription protein (STAT) and vascular endothelial growth factor (VEGF) signaling pathways. At the same time, they were significantly enriched in cytoplasmic retinoic acid-inducible gene I (RIG-I)-like receptor (RLR) signaling pathway, Influenza A, TNF signaling pathway, cytosolic DNA-sensing pathway, nucleotide-binding and oligomerization domain (NOD)-like receptor (NLR) signaling pathway, and HSV-1 infection (**Figure 5A** and **Supplementary Table 2**) (15). The RIG-I and melanoma differentiation-associated gene 5 (MDA5) proteins, encoded by DEXH (Asp‐Glu‐X‐His) box polypeptide 58 (*DDX58*) and IFN-induced helicase C domain-containing protein 1 (*IFIH1*) respectively, are both important dsRNA recognition receptor in the RLR signaling pathway, and BTV infection induced transcriptional level of *IFIH1* was higher than that of *DDX58* in our study (**Figure 3A** and **Supplementary Table 2**) (50). In addition to being assigned in typical dsRNA sensing signal pathway, dif-mRNAs were also enriched in DNA virus infection-related signaling pathways, such as Herpes simplex virus 1 (HSV-1) infection, Epstein-Barr virus (EBV) infection and Kaposi sarcoma-associated herpesvirus (KSHV) infection (**Figure 5A**) (50). Using the HSV-1 pathway as an example, 45 dif-mRNAs were involved, with 25 dif-mRNAs being transcriptionally increased and 20 dif-mRNAs being transcriptionally decreased. The 25 upregulated dif-mRNAs contained not only typical dsRNA activated genes, such as Toll/IL-1 receptor domain-containing adapter molecule 1 (*TICAM1*, also known as *TRIF*), an adapter of TLR3, but also traditional DNA recognition receptor, for instance, Toll-like receptor 9 (*TLR9*) (**Supplementary Table 2**) (51, 52). The 20 decreased dif-mRNAs mainly were zinc finger proteins relevant to DNA binding and transcription (**Supplementary Table 2**) (53).

The target genes of dif-miRNAs mainly were associated with the lysosome, gap junction, Influenza A, transcriptional misregulation in cancer, apoptosis, and adenosine 5’-monophosphate-activated protein kinase (AMPK) signaling pathway (**Figure 5B** and **Supplementary Table 6**). Previous studies have shown that apoptosis triggered by BTV infection in mammalian cells is the leading cause of CPE/cell lysis *in vitro* and virus-induced pathogenesis *in vivo* (9, 54, 55). The target genes of dif-miRNAs, calcium-activated neutral proteinase 1 (*CAPN1*), a requirement of apoptosis in some cell lines, and caspase-7 (*CASP7*), served as one of the readouts for the activation of apoptosis, were both transcriptionally upregulated by BTV-1 infection. On the other hand, downregulated dif-miRNAs, hsa-mir-5100-p3_1ss18TC, and chi-miR-195-3p_R+2, possibly contributed to the expression increases of the two genes mentioned above (**Figure 3B** and **Supplementary Table 6**) (56). The dif-lncRNAs targeting genes were primarily involved in the γ-aminobutyric acid (GABA)ergic synapse, antigen processing and presentation, D-Glutamine and D-glutamate metabolism, Influenza A, inflammatory bowel disease (IBD) and NLR signaling pathway (**Figure 5C** and **Supplementary Table 7**). In addition, most linear transcripts of dif-circRNAs were related to the central carbon metabolism in cancer, D-Glutamine and D-glutamate metabolism, renal cell carcinoma, glycosphingolipid biosynthesis-lacto and neolacto series, arginine biosynthesis and colorectal cancer (**Figure 5D** and **Supplementary Table 8**). These results showed that except for virus-sensing and signaling transduction pathways, the dif-RNAs induced by BTV-1 infection also involve in a variety of pathways, such as cancer-, transcription-, and metabolism-related pathways, indicating that BTV can regulate the expression profiles of OA3.Ts cells and hijacks the metabolic pathways of the host in order to complete its life cycle.

### ceRNA networks construction and enrichment analyses

**Figure 6:**
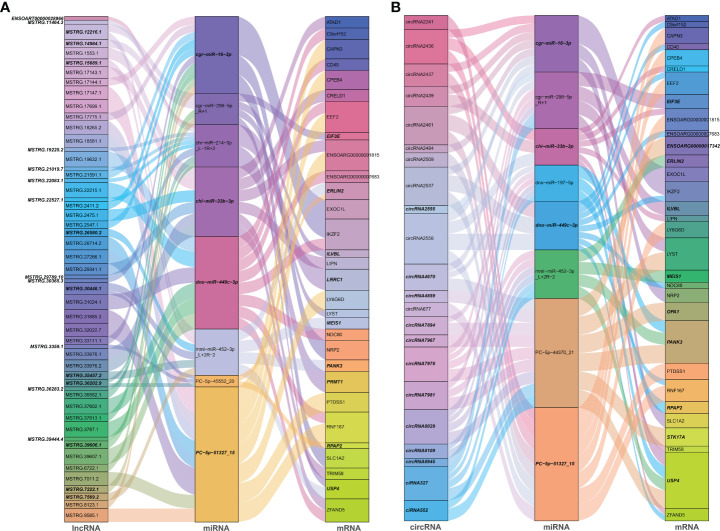
Sankey diagrams of ceRNA network based on **(A)** lncRNA-miRNA-mRNA, and **(B)** circRNA-miRNA-mRNA interaction pairs. Different colored blocks represented different dif-RNA nodes, and the downregulated dif-RNAs were indicated with bold italic font. The flow charts from dif-lncRNAs or dif-circRNAs to dif-miRNAs, and finally to dif-mRNAs represented lncRNAs or circRNAs regulated the expression of mRNAs through different miRNAs.

Noncoding RNAs (ncRNAs) involve a wide range of regulatory functions that interact with DNA and RNA to control transcription and translation. In addition, lncRNAs and circRNAs possess miRNA binding sites, which allow them to compete for binding miRNAs, serve as miRNA sponges, and counteract the repressive transcriptional activity of miRNA on target genes, thereby achieving indirect regulation of gene expression. These lncRNAs and circRNAs are called ceRNAs (13, 14). Based on the ceRNA theory, lncRNA-miRNA-mRNA and circRNA-miRNA-mRNA ceRNA networks were constructed, using lncRNAs or circRNA as the decoy, miRNA as the core, and mRNA as the target. According to the screening criteria, 53 dif-lncRNAs and 22 dif-circRNAs regulating 33 dif-mRNAs mainly through 10 dif-miRNAs were selected for display (**Figures 6A, B** and **Supplementary Table 9**). The heatmaps depicted the expression patterns of partial dif-RNAs in the infected and mock-infected groups (**Figures 3A–D**).

**Figure 7:**
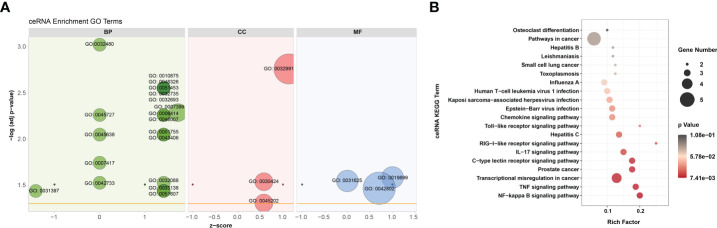
GO annotation and KEGG enrichment of dif-mRNAs involved in ceRNA networks. **(A)** Z-score in the GO bubble diagram was equal to the total number of transcriptional increased dif-mRNAs minus the total number of decreased dif-mRNAs and then divided by the square root of the total number of dif-mRNAs in a certain GO term and was used to measure whether the term was upregulated or downregulated overall. The size of the solid circulars represented the number of enriched dif-mRNAs under specific GO terms. GO: 0032480: negative regulation of type I interferon production, GO: 0045727: positive regulation of translation, GO: 0045638: negative regulation of myeloid cell differentiation, GO: 0007417: central nervous system development, GO: 0042733: embryonic digit morphogenesis, GO: 0031397: negative regulation of protein ubiquitination, GO: 0010875: positive regulation of cholesterol efflux, GO: 0046326: positive regulation of glucose import, GO: 0051453: regulation of intracellular pH, GO: 0032735: positive regulation of interleukin-12 production, GO: 0032693: negative regulation of interleukin-10 production, GO: 0007399: nervous system development, GO: 0006414: translational elongation, GO: 0046007: negative regulation of activated T cell proliferation, GO: 0001755: neural crest cell migration, GO: 0043406: positive regulation of MAP kinase activity, GO: 0032088: negative regulation of NF-kappaB transcription factor activity, GO: 0033138: positive regulation of peptidyl-serine phosphorylation, GO: 0051607: defense response to virus, GO: 0032991: protein-containing complex, GO: 0030424: axon, GO: 0045202: synapse, GO: 0019899: enzyme binding, GO: 0031625: ubiquitin protein ligase binding, GO: 0042802: identical protein binding. **(B)** Top 20 KEGG pathways were selected to display. Rich factor indicated the ratio of dif-mRNAs enriched to the specific pathway. The size of and the color of the solid circulars represented the number of enriched dif-mRNAs and significance of the enrichment, respectively.

According to GO analysis, the regulated genes in the ceRNA networks are primarily enriched in BP and CC ontologies, such as the negative regulation of IFN-I production (GO: 0032480), positive regulation of cholesterol efflux (GO: 0010875), positive regulation of glucose import (GO: 0046326), regulation of intracellular pH (GO: 0051453), positive regulation of IL-12 production (GO: 0032735), and protein-containing complex (GO: 0032991) (**Figure 7A** and **Supplementary Table 9**). Ubiquitin-specific protease (USP4), a key modulator of RLR signaling pathway, increases the stability of RIG-I protein through deubiquitination and further promotes the production of IFN-I (57). BTV-1 infection downregulated a series of lncRNAs and circRNAs, such as MSTRG.19225.2 and circRNA7978, and probably deprived their competitive binding with the corresponding miRNAs, cgr-miR-298-5p_R+1, and ultimately led to a certain extent of decrease in the transcription of *USP4* (**Figures 6A, B**). KEGG pathway analysis indicated that the modulated target genes are mostly assigned to the NF-κB signaling pathway, TNF signaling pathway, transcriptional misregulation in cancer, prostate cancer, C-type lectin receptor signaling pathway, and IL-17 signaling pathway (**Figure 7B** and **Supplementary Table 9**). Except for transcriptional misregulation in cancer, IκBα was mentioned to function in the above 5 pathways (**Supplementary Table 9**) (47, 52, 58). Several significantly elevated lncRNAs and circRNAs, MSTRG.37789.1, MSTRG.29725.1, circRNA2437, and circRNA2439, served as sponges of hsa-mir-7977-p5_1ss5AG, and contributed to the increased transcription level of *NFKBIA*. The GO and KEGG enrichment analyses suggested that most dif-mRNAs in the ceRNA networks are associated with the host’s inflammatory responses, such as IL-12-, IL-17-, and TNF-related biological processes or signaling pathways (52, 59, 60).

### Protein-protein interaction network, modules extraction, and functional enrichment analyses

**Figure 8:**
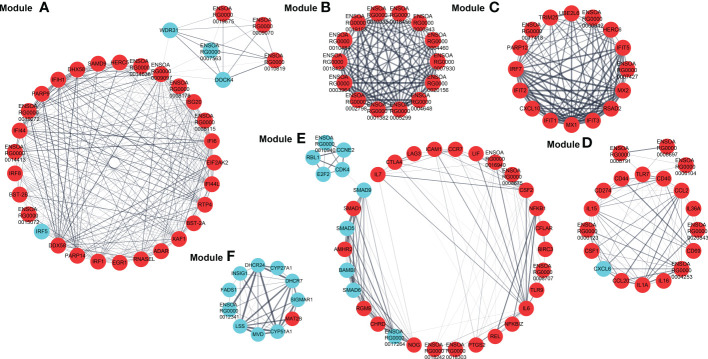
Modules **(A–F)** extracted from PPI network. The MCODE plugin in Cytoscape was applied to extract densely connected modules from PPI network, with degree cut-off = 2, node score cut-off = 0.2, K-score = 2, and max depth = 100. Red and blue circles represented upregulated and downregulated dif-mRNAs, respectively, whereas the thickness of the solid black line indicated the strength of the corresponding protein interactions.

The PPI network based on dif-mRNAs consisted of 1306 nodes and 7052 interaction pairs (**Supplementary Figure 1**). In the PPI network, six modules were determined, which were mainly related to viral nucleic acids sensing, antiviral effects, inflammatory responses, development, cell cycle (Module A, C, D and E), ribosome structural constituent proteins (Module B), and cholesterol and steroid metabolism process (Module F), respectively (**Figure 8**).

**Figure 9:**
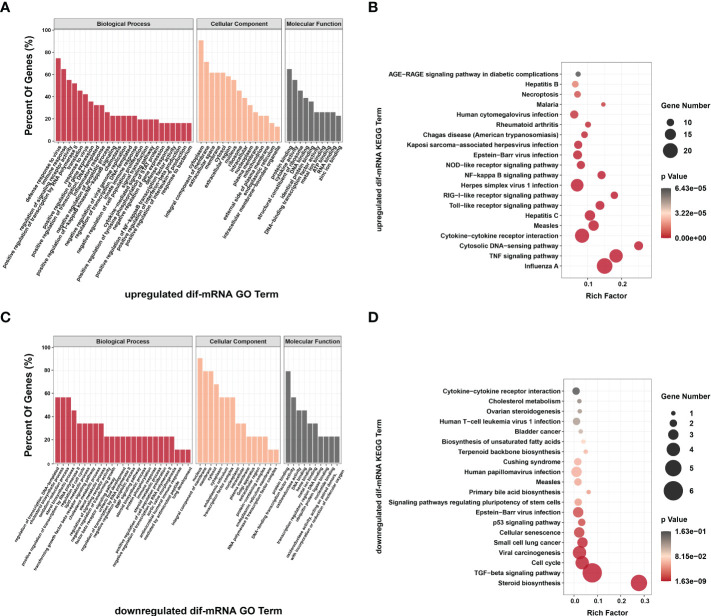
Go annotation and KEGG enrichment of upregulated and downregulated dif-mRNAs displayed in the extracted six modules. Top 50 GO terms and top 20 KEGG pathways were shown. **(A, C)** The height of bar chart represented the ratio of enriched dif-mRNAs under specific GO terms. **(B, D)** Rich factor of the KEGG scatter diagrams indicated the ratio of dif-mRNAs enriched to the specific pathway. The size of and the color of the solid circulars represented the number of enriched dif-mRNAs and significance of the enrichment, respectively.

Furthermore, dif-mRNAs in these modules were extracted and performed for GO enrichment and KEGG analyses (**Supplementary Tables 10–13**). Functional enrichment analysis showed that according to the numbers of enriched dif-mRNAs, the elevated dif-mRNAs are primarily relevant to defense response to virus, immune response, cytoplasm, integral component of membrane, extracellular space, and protein binding under BP, CC and MF ontologies, respectively (**Figure 9A** and **Supplementary Table 10**). In contrast, the declined dif-mRNAs were mainly associated with regulation of transcription (DNA-templated), cholesterol biosynthetic process, nucleus, integral component of membrane, membrane, and protein binding under BP, CC and MF ontologies, respectively (**Figure 9B** and **Supplementary Table 11**). Taking Module A as an example, it contained 35 nodes and 284 interaction pairs, including expression-increased *DDX58*, *IFIH1*, *ISG20*, IFN-α inducible protein 6 (*IFI6*), IFN-induced protein 44-like (*IFI44L*), bone marrow stromal cell antigen 2A (*BST-2A*), *BST-2B*, IFN regulatory factor 1 (*IRF1*), and ribonuclease L (*RNASEL*) in defense response to virus term under BP ontology, and transcriptional-elevated *ENSOARG00000019675* and *ENSOARG00000010819* in ribosome term under CC ontology (**Figure 8**) (15, 50, 61–65).

The top 20 KEGG pathways for upregulated and downregulated dif-mRNAs in the modules were chosen for display in the order of significance. The upregulated dif-mRNAs primarily enriched in Influenza A, TNF signaling pathway, cytosolic DNA-sensing pathway, cytokine-cytokine receptor interaction, Measles, and Hepatitis C (**Figure 9C** and **Supplementary Table 12**). The downregulated dif-mRNAs mainly enriched in steroid biosynthesis, TGF-β signaling pathway, cell cycle, viral carcinogenesis, small cell lung cancer, and cellular senescence (**Figure 9D** and **Supplementary Table 13**). Steroids and steroid hormones function in metabolism, stress responses, immune activities, sexual differentiation and reproduction, and several genes related to steroid biosynthesis were decreased significantly in BTV-1-infected OA3.Ts cells, such as lanosterol synthase (*LSS*), cytochrome P450 lanosterol 14-α demethylase (*CYP51A1*), 7-dehydrocholesterol reductase (*DHCR7*), and *DHCR24* (**Figure 8**) (66). Moreover, the transcription level of another member of the cytochrome P450 family, *CYP11A1*, also declined (**Supplementary Table 2**). CYP11A1 is the first and rate-limiting enzyme in steroidogenesis and converts cholesterol to pregnenolone, the precursor of steroid hormones (67). Pregnenolone further produces 17α-hydroxypregnenolone, dehydroepiandrosterone, androstenedione, and testosterone under the catalysis of enzymes, which plays an important role in spermatogenesis (68). Downregulated genes, mothers against decapentaplegic homolog 5 (*SMAD5*), *SMAD6*, and *SMAD9*, showed in Module E, are all associated with TGF-β signaling pathway, which involves cell fate, proliferation, terminal differentiation, and cell death (**Figure 8**) (69). BTV caused repression of this pathway by reducing the expression of cell cycle-related genes, such as cyclin E2 (*CCNE2*), cyclin-dependent kinase 4 (*CDK4*), S-phase kinase associated protein 2 (*ENSOARG00000019040*), and retinoblastoma transcriptional corepressor like 1 (*RBL1*), and might further lead to cell cycle arrest in infected OA3.Ts cells (**Figure 8**) (70).

### Validation of dif-RNAs using qRT-PCR and WB

**Figure 10:**
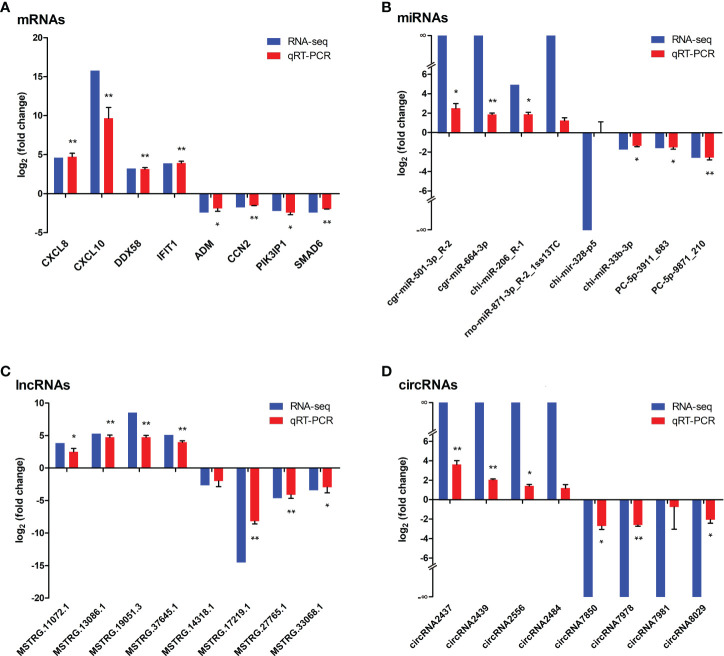
Verification the expressions of **(A)** dif-mRNAs, **(B)** dif-miRNAs, **(C)** dif-lncRNAs, and **(D)** dif-circRNAs using qRT-PCR, respectively. Blue and red bars represented the whole transcription sequencing results and qRT-PCR validation results respectively, whereas qRT-PCR validation results were displayed as mean log_2_ (fold change) ± SD with error bars from three independent experiments. Symbols * and ** indicated the difference between the indicated groups was significant (*p*-value < 0.05) or very significant (*p*-value < 0.01 respectively.

Eight each of dif-mRNAs, dif-miRNAs, dif-lncRNAs, and dif-circRNAs were selected for qRT-PCR verification. As shown in **Figure 10**, the expression trends of the vast majority of selected dif-RNAs were highly consistent with the sequencing results, with significant differences between the infected and mock-infected groups, except for rno-miR-871-3p_R-2_1ss13TC, chi-mir-328-p5, MSTRG.14318.1, circRNA2484, and circRNA7981. In addition, some validated dif-RNAs in the infected and mock-infected groups were labeled in the heatmaps, respectively (**Figures 3A–D**).

**Figure 11:**
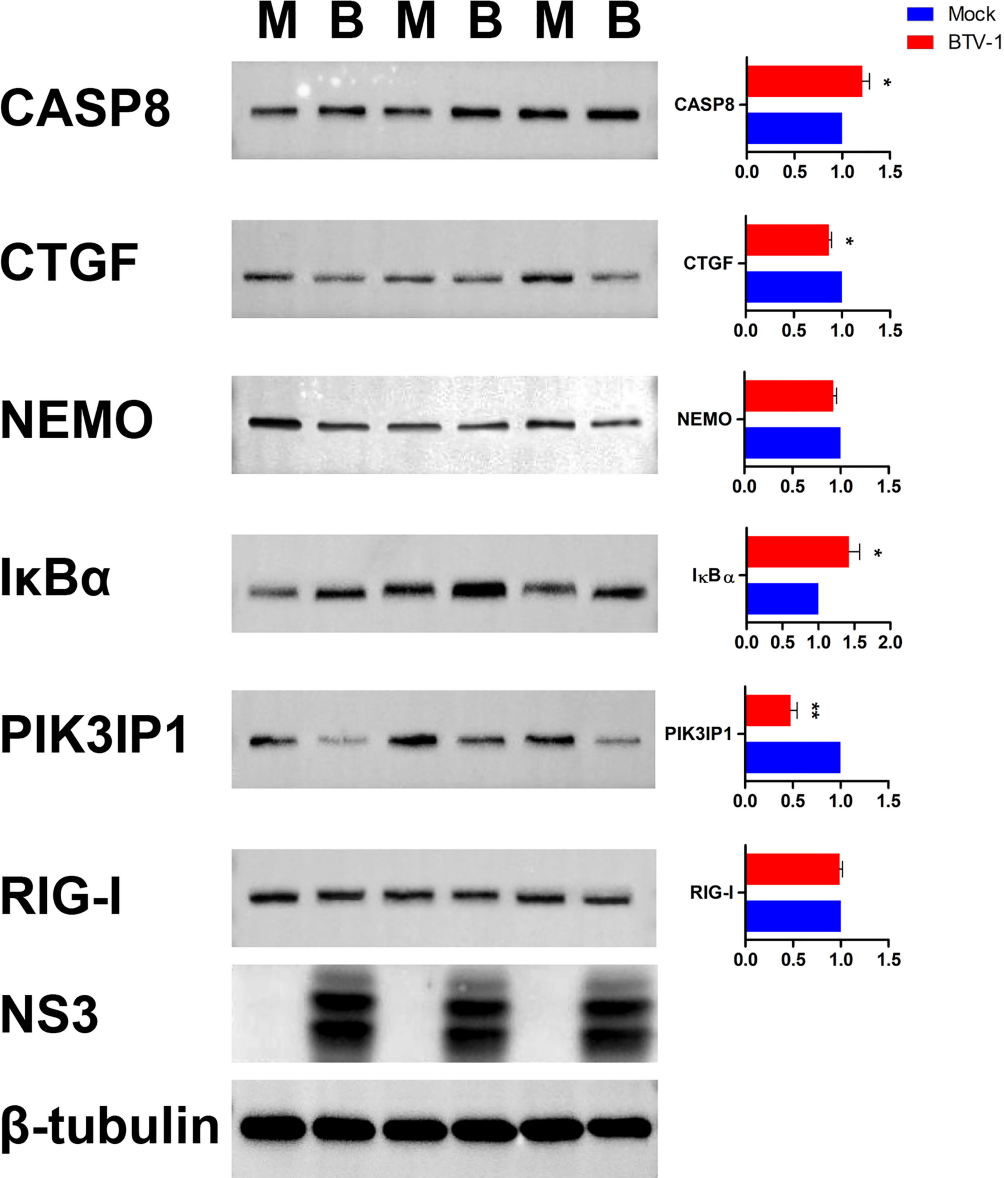
Validation the expressions of six dif-mRNAs using WB. The expression patterns of CASP8, CTGF, NEMO, IκBα, PIK3IP1, and RIG-I were validated using WB with specific antibodies. M and B indicated mock-infected and BTV-1-infected groups, respectively. NS3 was detected as control for successful infection, whereas β-tubulin served as an internal control. Symbols * and ** indicated the difference between the indicated groups was significant (*p*-value <0.05) or very significant (*p*-value <0.01), respectively.

Six proteins CASP8, connective tissue growth factor (CTGF, also known as CCN2), NEMO, IκBα, PI3K interacting protein 1 (PIK3IP1), and RIG-I were selected for further WB validation. Except for RIG-I, the expression patterns of other proteins coincided with the sequencing results (**Figure 11**).

## Discussion

BTV pathogenicity and its interactome with the host are highly complex. Moreover, alternations in the host transcription profiles caused by BTV infection are enormous and systemic. However, the RNA expression profiles and involved biological processes in host cells responding to BTV infection have not been comprehensively elucidated. As an increasingly popular method to detect genome-wide gene expression, the combination of expression profile data and bioinformatics analyses has become an effective modality for identifying potential biomarkers and key pathways in various pathogens’ infections. In this study, in order to gain some clues for an in-depth understanding of BTV infection and pathogenic relevant mechanisms, we determined the differential expression profiles of mRNAs, miRNAs, lncRNAs, and circRNAs in OA3.Ts cells infected with BTV-1 through high-throughput whole-transcriptome sequencing, analyzed the GO terms and signaling pathways enriched by dif-RNAs, predicted the potential interactions between dif-RNAs, and verified the sequencing results of some dif-RNAs using qRT-PCR and WB.

BTV regulates the transcription system, hijacks biological pathways, and utilizes factors of the mammalian host to complete its life processes, such as infection, replication, packaging, and release, which can lead to a series of reactions in the host cells, including but not limited to autophagy, apoptosis, and cell lysis (9, 54, 71, 72). These reactions are a wrestle between BTV and the host cells, progressively advanced and completed. Therefore, some critical time nodes are significant to completing the virus life cycle. As observed in **Figure 1A**, previous studies also pointed out that the autophagy and apoptosis phenomenon gradually appeared from 24 hpi (10, 11, 71). In addition, the IFN levels decreased and became undetectable at 24 hpi (55); As for reversible protein phosphorylation, the most pervasive control and regulatory mechanism within cells, also began to transit from 24 hpi, for example, the phosphorylation status of Akt exhibited a significant conversion from increasing to decreasing at 24 hpi (73). Hence, we selected OA3.Ts cells infected with BTV-1 for 24 h as samples for whole-transcriptome sequencing to clarify the transcription profiles changes induced by BTV infection.

Although human lung adenocarcinoma (A549) cells were thoughted to be a good model for studying BTV infection, our sequencing data identified relatively few dif-mRNAs compared to the 2863 dif-mRNAs previously found between BTV-8-infected and mock-infected A549 cells. While a comparable number of dif-mRNAs was obtained in our study in comparation with 1152 dif-mRNAs determined in research conducted in BTV-16-infected sheep PBMCs (**Supplementary Table 2**) (12, 15). There was no doubt that A549 cells and OA3.Ts cells derived from different species is one reason for the differences, whereas another more important reason was that the BTV-8 strain with stronger virulence and higher MOI of 4 was used to infect A549 cells and induced more dramatic host responses, which ultimately manifested as extensive changes in the expression of genes. Although the same serotype strains were used to infect sheep-derived cell lines, the highly tissue-specific expression of some miRNAs and the difference in infection time may lead to divergences in miRNA expression profiles, similar to the changes caused by other RNA viruses (74, 75). Therefore, only 9 dif-miRNAs were shared between the 78 dif-miRNAs identified in our study and the 265 dif-miRNAs obtained in the previous study, and merely 6 dif-miRNAs possessed similar expression patterns (**Supplementary Table 3**) (10).

Through functional analyses, it was found that a total of 35 dif-mRNAs are enriched in the defense responses to virus term (GO: 0051607), which involved RLR signaling pathway, TLR signaling pathway, and NLR signaling pathway (**Figures 4A**, **5A** and **Supplementary Table 2**). RLR and TLR signaling pathways are primarily responsible for sensing viral RNAs (50). RIG-I and MDA5 act as dsRNA sensors within the RLRs family and recognize relatively short and long dsRNAs, respectively, which explains why the expression level of *IFIH1* encoding MDA5 induced by BTV infection was higher than that of *DDX58* encoding RIG-I (**Supplementary Table 2**) (52, 76). Despite the role of LGP2, another member of the RLRs family encoded by DEXH (Asp‐Glu‐X‐His) box polypeptide 58 (*DHX58*), in resisting RNA virus infection is not yet fully understood, the transcriptional level of *DHX58* was upregulated, although the fold change was lower than those of the other two members (**Supplementary Table 2**). The same expression pattern of *DDX58*, *DHX58*, and *IFIH1* was also detected in BTV-8-infected A549 cells (12). TLR3, TLR7, and TLR9 of the TLRs family sense dsRNA, single-stranded RNA, and DNA, respectively. Despite the previous study pointing out that BTV-activated IFN-I responses *via* TLR3 recognition, the expression level of TLR3 was not induced by BTV infection in our study (77). Instead, the expression levels of TLR7 and TLR9 were significantly increased, which were partially consistent with the previous studies (**Supplementary Table 2**) (12, 15). Nevertheless, whether the TLR signaling pathway is activated by virus infection cannot be generalized. For instance, Chauveau et al. (2012) indicated that impairment of TLR3 exhibits no or weak effect on IFN-I transcription induced by BTV-8; Singh et al. (2017) regarded TLR7 as an important immune gene for resisting BTV-16 infection in sheep PBMCs; Ruscanu et al. (2012) noted that BTV-8 induces IFN-I production in sheep plasmacytoid dendritic cells (DCs) *via* a TLR7-independent signaling pathway; As for the TLR9-related signaling pathway, a typical DNA sensing pathway, could be activated by the dengue virus in human DCs (15, 76, 78, 79). Hence, whether TLRs are activated depends on the virus species and the type of cells it infects, and the transcription levels of TLRs induced by BTV infection might also be relevant to cell types.

In response to BTV infection, RNA virus and DNA virus sensing receptor and adaptor proteins showed a degree of versatility, and their downstream signal transmitters present extensive crosstalk to save resources and improve the efficiency of the host (80). Just like Z-DNA binding protein 1 (*ENSOARG00000017418* also known as *ZBP1*) and cyclic GMP-AMP synthase (*ENSOARG00000006104* also known as *cGAS*), which play important roles in restricting RNA and DNA virus infections, were all transcriptionally elevated obviously in our study (**Supplementary Table 2**) (80, 81). Furthermore, RNA virus infection plausibly prompts DNA damage in the host cell, and the leaked self-DNA further activates DNA recognition pathways and increases the production of IFNs and inflammatory cytokines (82). Thus, it was not unexpected that dif-mRNAs induced by BTV infection were enriched in HSV-1, EBV and KSHV infection related signaling pathways (**Figure 5A** and **Supplementary Table 2**).

The final result of recognizing viral DNA or RNA is the production of IFNs, the core effector of the host’s innate immune system. Thus, the confrontation between BTV and the innate immune system was reflected in both sides’ modulation of IFN-I production. Aside from *USP4*, some other ubiquitin transferases, such as RING-type E3 ubiquitin transferase (*RNF125*) and SMAD ubiquitylation regulatory factor 1 (*SMURF1*), which are negative regulators of RIG-I, MDA5, as well as mitochondrial antiviral-signaling (MAVS), were significantly induced expression by BTV (**Supplementary Table 2**) (83). As a counterattack from the host, tripartite motif 25 (*TRIM25*), an indispensable positive regulator of RIG-I, was also obviously upregulated, which functions to activate the activity of RIG-I. However, the extent of the increase was slightly inferior to that of *RNF125* (**Supplementary Table 2**) (83). Our sequencing data also revealed that in contrast to the increased expression of RLRs family members, transcription of *MAVS* and tumor necrosis factor receptor-associated factor 3 (*TRAF3*) within the RLR signaling pathway did not change significantly, potentially further weakening signal transduction downstream. Furthermore, the downregulation of *IKBKE* and *IKBKG* could cause the profound interruption of signal transduction and direct impairment of IFN-I production through IRF3 (**Supplementary Table 2**) (52, 84). Nevertheless, the transcription of other important regulators that negatively modulate IFN-I production by targeting RIG-I downstream proteins and NEMO, such as *TRIM18* and *TRIM29*, showed no significant concomitant changes (85–87). In order to defeat BTV, the host cells also arranged other trumps. Poly(ADP-ribose) polymerases (PARPs) modulate the innate immune responses through recognition of viral RNAs, inhibiting viral transcription and replication, degradation of viral proteins, and facilitating transcription of *ISGs* to defend against virus invasion (88). In our study, the expression of *PARP9*, a non-canonical sensor for RNA virus, along with its binding partner, Deltex E3 ubiquitin ligase 3L (*DTX3L*), were all elevated; moreover, *PARP10*, *PARP12*, and *PARP14* were concomitantly upregulated significantly (**Supplementary Table 2**) (88, 89). The target genes of dif-lncRNAs were also responsible for the enrichment in protein ADP-ribosylation (GO: 0006471) and nicotinamide adenine dinucleotide (NAD) + ADP-ribosyltransferase activity (GO: 0003950) terms under BP and MF ontologies (**Figure 4C** and **Supplementary Table 7**). In conclusion, despite BTV intensively regulated IFN-I production, it was still a strong IFN-I inducer. ISG15 is an IFN-induced expression protein that mediates ISG15ylation by conjugating itself with other proteins and causing the degradation of corresponding proteins (90). The transcription levels of homologous to E6AP carboxyl terminus (HECT) domain and regulator of chromosome condensation 1 (RCC1)-like domain-containing protein 5 (*HERC5*) and *HERC6*, which are associated with ISG15ylation, were all elevated significantly (**Supplementary Table 2**) (90). Similarly, *HERC5* was upregulated in BTV-16-infected sheep PBMCs and functioned as a critical immune gene (15).

The NLR signaling pathway, the third pathway involved in defense responses to virus, mainly recognizes invading bacteria and regulates the host’s inflammatory responses (52, 91). Our study did not observe alternations of important sensors in NLR signaling pathway, such as NOD1 and NOD2, contrary to the study conducted in BTV-16-infected sheep PBMCs (**Supplementary Table 2**) (15). At the same time, the expression of other members of the NLRs family was significantly changed, just like increases of NLRs family pyrin domain-containing 3 (*NLRP3*) relevant to inflammasome formation. Elevation of caspase-1-like protein (*ENSOARG00000003068*), which belongs to the mammalian inflammatory caspase family and catalyzes the maturation of proinflammatory cytokine IL-1β, was also accompanied (Supplementary Table 2) (91). In addition to the abovementioned members of the NLRs family, the enrichment in the TNF, IL-12, and IL-17 production or signaling pathways, the high expression of *CXCL8* and *CXCL10*, and the upregulation of *IL-6* and prostaglandin-endoperoxide synthase 2 (*PTGS2*) positively contributed to the inflammatory responses of the host (**Figures 5**, **7**, and **Supplementary Table 2**) (8, 92). However, pro-inflammatory cytokines are a double-edged sword. On one side, they control the scope of viral infection. On the other side, they also contributed to cytokines storm, capillary vessels damage and increased vascular permeability, coupled with the transcriptionally enhanced vascular endothelial growth factor (*VEGF*) and vascular endothelial growth factor receptor 2 (*KDR*), which would eventually lead to an aberrant systemic inflammatory response and severe hemorrhages in infected animals (**Supplementary Table 2**) (8, 92, 93).

In this still stalemate war, the virus has already narrowly won some important battles, such as transcription and translation regulations, cell proliferation and differentiation, metabolism, autophagy, and apoptosis. Seventy dif-mRNAs were enriched in DNA-binding transcription factor activity (GO: 0003700) term under MF ontology, and the expression of several essential transcription factors was downregulated. The transcription of a considerable amount of zinc finger proteins was also reduced (**Figure 4** and **Supplementary Table 2**). The general elevation of dif-mRNAs encoding ribosome constitutive proteins exhibited in **Figure 8** Module B suggested that BTV hijacks the host’s translation system to synthesize viral proteins (94). Given that the dysregulation of transcription and translation is bound to influence the proliferation and differentiation of host cells, the expression declined *SMAD6* and *SMAD9* were the direct executors of inhibiting these processes (**Figure 8** and **Supplementary Table 2**) (69). Additionally, there was a widespread transcriptional reduction of cholesterol and steroid metabolism-relevant genes (**Supplementary Table 2**), which might be the underlying mechanism behind the phenomenon of testicular degeneration and azoospermia in BTV-infected rams described in a previous study (2). Similarly, BTV infection elicited wide-ranging transcriptional misregulation of dif-miRNAs and dif-circRNAs enriched in cancer-related pathways (**Figures 5B, D**; **Supplementary Tables 6**, **8**). Glutamine, a crucial extracellular carbon source, is relied upon to replicate of various viruses (95, 96). BTV also modulated the D-glutamine and D-glutamate metabolism in infected OA3.Ts cells through dif-lncRNAs and dif-circRNAs (**Figures 5C, D**; **Supplementary Tables 7**, **8**). In a word, the abnormal regulation of transcription, translation, and metabolism by BTV inevitably caused various dysfunction in the host cells.

Talking about the slightly superior victory of BTV, we have to mention autophagy and apoptosis. Because, on one hand, although the host activates autophagy and apoptosis to clear damaged organelles and infected cells, limiting the spread of virus. It also carefully controls the extent of autophagy and apoptosis to mitigate the self-damage. On the other hand, the viruses enhancing autophagy and apoptosis to complete their life cycle are their customary means (11, 71). As observed in **Figure 1**, BTV infection had prompted apoptosis steadily, and the visible CPEs gradually expanded from 24 hpi to 48 hpi. Moreover, apoptosis implicates the ordered function of multiple proteases, including peptidase, hydrolase, and endopeptidase (97). The proteases CASP8 and CASP7, activator and executor of apoptosis, were inductively highly transcribed (**Supplementary Table 2**) (55). Matrix metalloproteinases (MMPs) are proteolytic enzymes that cleave almost all extracellular matrix components. MMP1, MMP3, MMP12, and MMP13 served as collagenase, stromelysins, and elastase, respectively, and the genes encoding these four proteases were upregulated in our study, which would change the extracellular environment and exacerbate apoptosis (**Supplementary Table 2**) (98). At the same time, dif-miRNAs also appreciably contributed to the positive regulation of apoptosis flux (**Figure 4B** and **Supplementary Table 6**). Additionally, the elevated autophagy related 2B (*ATG2B*), an indicator of late steps of autophagy, and other relevant dif-mRNAs all played positive roles in the eventual execution of autophagy (**Supplementary Table 2**) (99). Simultaneously, GO analysis indicated that there are about a dozen of dif-circRNAs that also probably make contributions to the regulation of autophagy (**Supplementary Table 8**).

Finally, we needed to investigate the NF-κB signaling pathway separately because it is involved in transcription regulation, inflammation, and apoptosis (11, 41, 100). In our study, the elevated transcription levels of *REL*, *NFKB1*, *RELB*, *TANK*, *IL-1α*, *IL-6*, and *CXCL8* suggested that canonical and non-canonical NF-κB signaling pathway were activated, at least before 24 hpi. This was consistent with the findings of Stewart et al. (2010) that the NF-κB signaling pathway was activated in the early stage of BTV infection, thereby inhibiting BTV replication (**Supplementary Table 2**) (11). From 24 hpi, the activity of canonical and non-canonical NF-κB signaling pathway was suppressed by BTV, as evidenced by increased transcription levels of their repressors, *NFKBIA* and *NFKBIB*, as well as decreased expression levels of *IKBKG* and *IKBKE* with the intermediary centrality in the NF-κB interactive network (**Supplementary Table 2**) (47). Although the expression levels of some downstream genes and facilitators remained high, just like *REL*, *NFKB1*, *RELB*, and *TANK*, these high levels of transcription were likely in vain relative to the downregulation of the betweenness center, and the upregulation of repressor genes since the anti-apoptotic function of the NF-κB signaling pathway was progressively lost starting from 24 hpi. Apoptosis and its resulting CPEs significantly expanded their influences at 48 hpi, the most common and best-characterized form of inflammation-induced cell death (**Supplementary Table 2**) (11, 47).

Our study presented a whole transcriptome map of BTV-infected sheep embryonic testicular cells for the first time, outlined the context of virus-and-host cell interactions, and provided new clues to elucidate the underly mechanisms of the host by which BTV exploited or hijacked. Unfortunately, the NF-κB complex interactive network involves more than 300 members, and it is difficult to identify its role in the BTV infection process from the current results. Our sequencing data could only suggest similar conclusions to previous studies: BTV activated NF-κB signaling pathway in the early stage of infection, but inhibited it in the late stage, and BTV-induced apoptosis did not depend on the activation of this signaling pathway (11). We speculate that other signaling pathways, similar to NF-κB pathway, are regulated differently in different stages of infection with the progression of BTV infection. At the same time, further transcriptome sequencing and experimental verification at more intensive infection time points will help us to understand the different regulations of these signaling pathway more clearly in the process of BTV infection.

The variation of the transcriptional profile implicated a series of transcription factors, and the changes in transcription factors and validation of transcriptomic sequencing data could be further analyzed in conjunction with an assay for transposase-accessible chromatin with high-throughput sequencing (ATAC-seq). The OA3.Ts cell is a sheep embryo-derived cell line with differentiation potential, resulting in a relatively complex cell type composition. The transcriptional profile changes involved in the BTV infection process vary widely among different type of cells, which may cause the expression of some transcripts to be annihilated, and then exert an impact on our analysis results. Single-cell transcriptional profiling is also a promising technology for elucidating the infection and pathogenic mechanisms of BTV at the transcriptional level.

## Data availability statement

The datasets presented in this study can be found in online repositories. The names of the repository/repositories and accession number(s) can be found below: https://www.ncbi.nlm.nih.gov/geo/, GSE213637, GSE213638.

## Author contributions

ZrL and HL conceived and designed the experiments. ZrL, PZ, ZY, HY, and ZhL performed the experiments. DL and ZyL analyzed the data. ZrL and DL wrote the paper. All authors contributed to the article and approved the submitted version.

## Funding

This study was supported by the Ten Thousand Talent for Youth Top-notch Talents of Yunnan Province (Project No. YNWR-QNBJ-2020-211), the Applied Basic Research Program of Yunnan Province (Project No. 2019FB041), the National Natural Science Foundation of China (Project No. 31802177), and the Special Fund for Agro-scientific Research in the Public Interest (Project No. 201303035).

## Conflict of interest

The authors declare that the research was conducted in the absence of any commercial or financial relationships that could be construed as a potential conflict of interest.

## Publisher’s note

All claims expressed in this article are solely those of the authors and do not necessarily represent those of their affiliated organizations, or those of the publisher, the editors and the reviewers. Any product that may be evaluated in this article, or claim that may be made by its manufacturer, is not guaranteed or endorsed by the publisher.

## Supplementary material

Supplementary Figure 1 The PPI network based on dif-mRNAs constructed using STRING online database which was consisted of 1306 nodes and 7052 interaction pairs. Red and blue circles represented upregulated and downregulated dif-mRNA, respectively, whereas the thickness of the solid black line indicated the strength of the corresponding protein interactions.
