# Supplementary figures and images for: Whole-transcriptome analyses of sheep embryonic testicular cells infected with the bluetongue virus
Source: Front Immunol. 2022 Dec 1;13:1053059. doi: 10.3389/fimmu.2022.1053059 (PMC9751015; doi:10.3389/fimmu.2022.1053059)

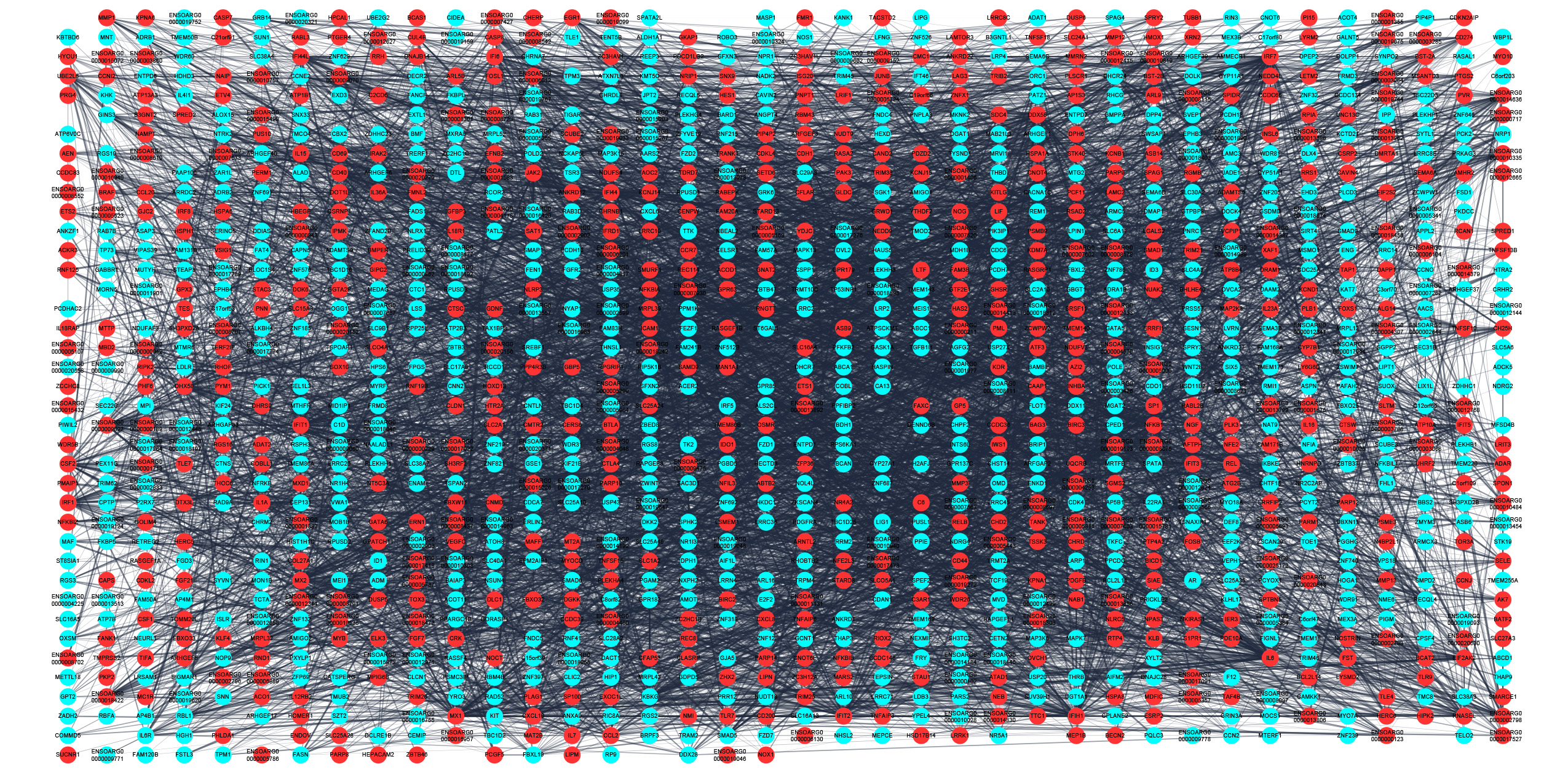

Supplement: Supplementary Figure 1 — The PPI network based on dif-mRNAs constructed using STRING online database which was consisted of 1306 nodes and 7052 interaction pairs. Red and blue circles represented upregulated and downregulated dif-mRNA, respectively, whereas the thickness of the solid black line indicated the strength of the corresponding protein interactions. [file Image_1.jpeg]
