# Supplementary material for: Whole-transcriptome analyses of sheep embryonic testicular cells infected with the bluetongue virus
Source: Front Immunol. 2022 Dec 1;13:1053059. doi: 10.3389/fimmu.2022.1053059 (PMC9751015; doi:10.3389/fimmu.2022.1053059)
Supplement: Supplementary file 2 [file Table_1.docx]

**Supplementary Table 1** Primers used in qRT-PCR validation of dif-RNAs

| **names of dif-RNAs** | **forward primer sequence (5'-3'）** | **reverse primer sequence** |
| --- | --- | --- |
| CXCL8 [1] | ACTGCGAAAATTCAGAAATCATTGTTA | CTTCAAAAATGCCTGCACAACCTTC |
| CXCL10 [2] | TCTAGGAACACACGCTGCAC | GACACGTGGGCAGGATTGAC |
| DDX58 [3] | CTTGCAAGAGGAATACCACTTAAACCCAGAGAC | TTCTGCCACGTCCAGTCAATATGCCAGGTTT |
| IFIT1 [4] | ACAGCAACCATGAGTTATAATGCT | GGTGCTTCACATAGGCCAGT |
| ADM [5] | ATGAAGCTGGTTCCCGTC | ACTTAGCGCCCATTTATTCCA |
| CCN2 (also known as CTGF) [6] | TGCACCAGCATGAAGACATACCGA | ACAGGAAGTGTGGTGGTTCTGTGA |
| PIK3IP1 | GCTGGAATCGTCCTTGGCTAC | GGACCACGACGGTCTTTTCA |
| SMAD6 | CAACCCCTACCATTTCAGCCG | AGAATTCACCCGGAGCTGTG |
| MSTRG.11072.1 | AGAATTAGTATGTCCGTTT | CTTTTCTACTTGAGCCAAT |
| MSTRG.13086.1 | GGCTGTAAACAATCCACAA | TATACTCTTCCGACATTACCAC |
| MSTRG.19051.3 | TCTCCGATACACAGAACGA | TGACTCTTTGACTGAGCAAG |
| MSTRG.37645.1 | GTACTATTTAATTGACGAGCTT | GCCCAAACATTCTTAACAGT |
| MSTRG.14318.1 | TTGTTATGTAGCATTATCACGG | GGACATTTGCTTACCCCTT |
| MSTRG.17219.1 | TAACTAGATGGACCTTAGTCG | AAAACATGGGAATATAGTCTG |
| MSTRG.27765.1 | ATAAGGGCTGATTCTATGTCC | CATTAAACCCCAGGAGTACGG |
| MSTRG.33068.1 | TAACCAGACAAATCGCTCCAC | CTCTTGCCTGGAAAATCCCATG |
| cgr-miR-501-3p_R-2 | AATGCACCCGGGCAAGGATTT |  |
| cgr-miR-664-3p | TATTCATTTACTCCCCAGCCTAC |  |
| chi-miR-206_R-1 | TGGAATGTAAGGAAGTGTGTGG |  |
| rno-miR-871-3p_R-2_1ss13TC | TGACTGGCACCACACTGGAT |  |
| chi-mir-328-p5 | TGTTTCGGAGCCTGGAGCG |  |
| chi-miR-33b-3p | CAGTGCCTCGGCAGTGCAGCC |  |
| PC-5p-3911_683 | CTGTACCACCTTGTCGGG |  |
| PC-5p-9871_210 | TTTAGCGCGCGCCTCTCAA |  |
| circRNA2437 | GAGTCGTCCTACCAAGCTGAA | AGGGTATGTGATTTAGTGTTGC |
| circRNA2439 | CCATTCGGGTGTCCAAAGTTGT | TCCCAGGCATAGATGGTCAG |
| circRNA2484 | TAAGAAGGCTCTGGCAAATG | GTACCACGGAGATGACTACTG |
| circRNA2556 | GGTCCAAATCTCAGCAACC | TGACTTGTATTGATCTGTCCTC |
| circRNA7850 | CAGTGCGTCTGGTCTTACGAG | CGATGTGTCTGAACCCTCACT |
| circRNA7978 | TGCCTTTTACGGACTTTGTT | CTAAATATGAACCAGCTCCAAC |
| circRNA7981 | TACAACAAAGTCATCGGGTGC | AGCCTCTCGGGTAAAGTTAGCA |
| circRNA8029 | TAAACAGAAATCACCTTCAGC | CCTCTGCAAATCCAATTCC |
| β-actin [1] | CTGAGCGCAAGTACTCCGTGT | GCATTTGCGGTGGACGAT |

[1] Karagianni AE, Vasoya D, Finlayson J, Martineau HM, Wood AR, Cousens C, et al. Transcriptional response of Ovine lung to infection with jaagsiekte sheep retrovirus. J Virol (2019) 93:e00876-19. doi: 10.1128/JVI.00876-19

[2] Wang Y, Han X, Zhang L, Cao N, Cao L, Yang L. Early pregnancy induces expression of STAT1, OAS1 and CXCL10 in Ovine spleen. Animals (2019) 9:882. doi: 10.3390/ani9110882

[3] Banerjee S, Pal A, Pal A, Mandal SC, Chatterjee PN, Chatterjee JK. RIG-I has a role in immunity against *Haemonchus contortus*, a gastrointestinal parasite in *Ovis aries*-a novel report. Front Immunol (2021) 11:534705. doi: 10.3389/fimmu.2020.534705

[4] Zhang R, Liu F, Hunt P, Li C, Zhang L, Ingham A, et al. Transcriptome analysis unraveled potential mechanisms of resistance to *Haemonchus contortus* infection in Merino sheep populations bred for parasite resistance. Vet Res (2019) 50:7. doi: 10.1186/s13567-019-0622-6

[5] Wu Y, Lin J, Li X, Han B, Wang L, Liu M, et al. Transcriptome profile of one-month-old lambs’ granulosa cells after superstimulation. Asian-Australas J Anim Sci (2017) 30: 20-33. doi: 10.5713/ajas.15.0999

[6] Morrison JL, Zhang S, Tellam RL, Brooks DA, McMillen IC, Porrello ER, et al. Regulation of microRNA during cardiomyocyte maturation in sheep. BMC Genomics (2015) 16:541. doi: 10.1186/s12864-015-1693-z
